# Rupture of Both Lungs by Wind of Bullet

**Published:** 1916-10

**Authors:** 


**Rupture of Both Lungs by Wind of Bullet.** Sencert, Rev. de Chir., Aug.-Nov., 1915, reports a case. The soldier died several hours later after repeated haemorrhages.

